# Classic Presentation of Catecholaminergic Polymorphic Ventricular Tachycardia: A Case Report

**DOI:** 10.7759/cureus.29844

**Published:** 2022-10-02

**Authors:** Emily E Hill, Amanda Schoonover, Christopher Benner, Todd P Chassee

**Affiliations:** 1 Human Medicine, Michigan State University, Grand Rapids, USA; 2 Emergency, Helen DeVos Children’s Hospital, Spectrum Health Medical Group, Grand Rapids, USA

**Keywords:** pediatric, emergency medicine, sudden cardiac death, sudden cardiac arrest, arrhythmia, syncope, catecholaminergic polymorphic ventricular tachycardia, case report

## Abstract

Syncope is a common reason for children and adolescents to seek care in the emergency department. Often syncopal episodes are benign and most commonly due to a vasovagal event. Occasionally an underlying cardiac arrhythmia is responsible. We present a case report of a 17-year-old male who collapsed during an emotional event and went into cardiac arrest. Emergency department evaluation including imaging, laboratory studies, and EKG indicated the cause of cardiac arrest was likely a primary cardiac arrhythmia. An initial clinical diagnosis of catecholaminergic polymorphic ventricular tachycardia (CPVT) was made based on symptom onset during an emotional event, family history of sudden cardiac death, patient age, past episodes of chest pain and palpitations, absence of structural heart defect, and lack of EKG changes after the return of spontaneous circulation (ROSC). The diagnosis was later confirmed with genetic testing. The patient was started on a beta-blocker and a subcutaneous implantable cardioverter-defibrillator (S-ICD, Boston Scientific, Marlborough, MA) was placed. Given the rarity of this condition, this diagnosis is often missed, which contributes to increased mortality rates. In children and young adults presenting with syncope without clear etiology in the presence of high-risk features, further evaluation should be performed including referral to cardiology to rule out chronic cardiac arrhythmias.

## Introduction

Syncope is a very common chief complaint in the Emergency Department (ED). It accounts for 1%-3% of ED visits annually [1,2]. In pediatric populations, up to 15% of patients will experience a syncopal event before the age of 18 [3]. This common event is not always benign. The possible etiologies of a syncopal episode include but are not limited to neurally mediated, cardiac, orthostasis, seizure, hypoglycemia, and most commonly idiopathic [2-6]. The most commonly known cause of syncope is vasovagal, representing 20%-40% of syncope cases, followed by orthostasis and cardiac at around 10% depending on the study [4-6]. Vasovagal syncope and orthostatic syncope are associated with low mortality, but cardiac etiology has a much higher mortality rate [2,4,5]. Our patient had a syncopal episode of cardiac etiology that resulted in cardiac arrest.

## Case presentation

The patient was a previously healthy 17-year-old who presented to the ED via emergency medical services (EMS) post-cardiac arrest. The patient was at home with his parents and engaged in a verbal argument when he had sudden onset of dyspnea and gasping. He grabbed his chest, and within moments became unresponsive and pale. The patient did not have a pulse and was not breathing. CPR was initiated by his parents, and EMS was contacted. Upon fire department arrival, CPR was continued, and an automated external defibrillator (AED) was placed. He was defibrillated two times before the return of spontaneous circulation (ROSC). The patient received CPR for a total of 20 minutes.

On arrival to the ED, the patient had a nasopharyngeal airway (NPA) in place with 2 liters of supplemental oxygen via nasal cannula, with oxygen saturations at 100%, pulse was 91 beats per minute, respiratory rate of 28, blood pressure of 111/58, and temperature of 36.4 ℃. On physical examination, the patient was ill-appearing with an NPA and cervical collar in place. He did not exhibit outward signs of trauma other than mild bruising to the neck noted by EMS. There was pink frothy sputum in the mouth. Breath sounds were coarse bilaterally, without wheezing, stridor, rhonchi, or rales. Heart rate was tachycardic with a regular rhythm. On neurological examination, the patient was unresponsive with a Glasgow Coma Score (GCS) of 6. He was able to move all four extremities and localize to pain. The patient did not have clonus and did not exhibit seizure-like activity. There was no evidence of tongue biting. Pupils were 6 mm, equal, round, and reactive to light.

**Figure 1:**
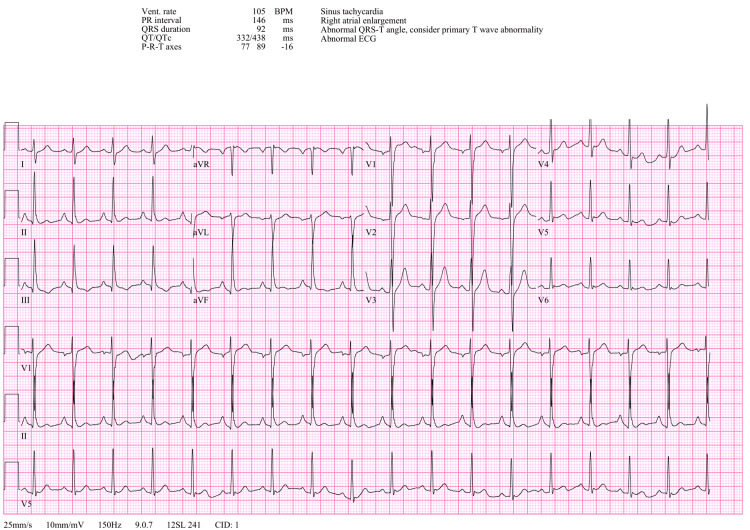
EKG on arrival to the ED.

Venous blood gas (VBG) was obtained and showed a pH of 7.23 and pCO_2_ at 59 mmHg. Given the patient’s respiratory acidosis and altered mental status with a GCS of 6, the decision was made to intubate the patient for airway protection. Initial differential included toxic ingestion, cardiac arrhythmia, status epilepticus, cerebral vascular accident, or metabolic dysfunction. Multiple laboratory studies were ordered as well as an electrocardiogram (EKG), chest x-ray, and CT of the head and neck. Significant findings included an elevated high sensitivity troponin of 166 (<22), elevated lactic acid of 5.0, and negative urinary drug screen, acetaminophen level, and salicylate level. EKG showed sinus tachycardia with normal PR interval at 146 ms and QTc interval at 438 ms. There was no evidence of acute ischemia or another worrisome arrhythmia (Figure 1). Imaging of the chest, head, and neck was unremarkable. Given the patient’s intubated status and recent cardiac arrest, he was admitted to the pediatric intensive care unit.

Once admitted, the patient’s neurologic exam continued to improve with spontaneous movements of the extremities. The patient did develop diastolic hypotension and therefore was placed on a norepinephrine drip and given a 2L normal saline bolus, with improvement. Pediatric cardiology and neurology were consulted to further elucidate the etiology of the patient’s cardiac arrest. An echocardiogram showed mild left ventricular systolic dysfunction with an ejection fraction of 54% and mild right ventricular systolic dysfunction but otherwise was structurally normal. Magnetic resonance imaging (MRI) of the brain was unremarkable. Prolonged video electroencephalography (EEG) showed generalized slowing that improved over the course of the study and mild discontinuity of the background over the first couple of hours which eventually disappeared. This was thought to be indicative of diffuse neuronal dysfunction which was nonspecific and may have been due to sedation. There were no focal deficiencies, no epileptiform discharges, and no seizures noted. The patient was kept in neurological protective measures. 

In pediatric cardiology’s discussion with the patient’s parents, it was discovered that the patient has had episodes in the past during emotional events where he grabbed his chest and needed to take deep breaths but had never experienced syncope. The patient’s paternal uncle had a sudden cardiac event at the age of 20 which resulted in death. Given the onset of cardiac arrest during an argument, prior episodes of chest pain and shortness of breath, family history of sudden cardiac death at a young age, absence of structural cardiac defect, and unremarkable EKG a clinical diagnosis of catecholaminergic polymorphic ventricular tachycardia (CPVT) was made. Genetic testing would be performed on an outpatient basis to confirm. A cardiac MRI and repeat MRI of the brain was ordered to rule out other possible etiologies.

The patient tolerated weaning ventilation settings and was extubated shortly thereafter. Post-extubation, the patient was awake, alert, and following commands. He had multiple episodes of agitation with short-term memory loss which required anxiolytics. Neurological examinations continued to improve, which made an ischemic brain injury unlikely. The repeat MRI of the brain was unremarkable. Cardiac MRI showed no evidence of prior myocardial infarction, acute myocarditis, or infiltrative cardiomyopathy. Left ventricular ejection fraction improved to 61% on follow-up echocardiogram. There were mild patchy opacities noted in bilateral lung fields thought to be due to aspiration pneumonitis or contusion secondary to chest compressions. 

Given the reassuring MRI of the brain and cardiac MRI, the patient was scheduled for subcutaneous implantable cardioverter-defibrillator (S-ICD, Boston Scientific, Marlborough, MA) placement and started on 40 mg nadolol daily. During placement, ventricular fibrillation was induced and successfully defibrillated with 65 joules. The patient had a short run of atrial fibrillation which was corrected back to normal sinus rhythm with multiple doses of esmolol.

Following the procedure, the patient’s continued difficulty with short-term memory and problem-solving skills were assessed on an outpatient basis. Speech-language pathology continued to evaluate the patient, and less than one month after discharge, cognitive and memory skills returned to the patient’s baseline.

**Figure 2:**
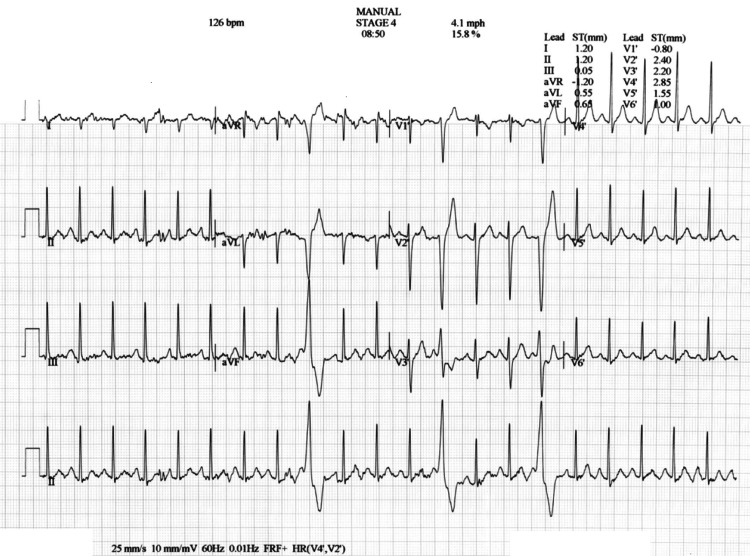
EKG from treadmill stress test showing singular polymorphic PVCs.

Genetic testing with an arrhythmia comprehensive panel revealed a pathogenic variant in the RyR2 gene, consistent with the autosomal dominant form of CPVT. During outpatient follow-up, a treadmill stress test revealed normal sinus rhythm at baseline, but premature ventricular contractions (PVCs) appeared at a heart rate of 126 bpm. These PVCs were singular, polymorphic, and disappeared with recovery (Figure 2). Given the PVCs, the patient’s daily dose of nadolol was increased to 60 mg QD. He continues to tolerate this dose well with no adverse side effects. Monthly transmissions from his S-ICD have shown a stable rhythm with no defibrillations delivered. 

## Discussion

This patient presented to the ED after an out-of-hospital cardiac arrest. The estimated incidence of sudden cardiac death (SCD) or sudden cardiac arrest (SCA) in children and young adults varies widely from 1.1 to 6.9 per 100,000 person-years, with a mortality rate reaching 89.6% [7-9]. Given the high mortality rate, further investigation has been done to evaluate the underlying etiology of the deaths. Channelopathies were determined to be the underlying pathology for 13% of patients after the autopsy, in patients ≤30 years of age, when there was not a known cause of the SCD [8]. The channelopathy determined to be the cause of this patient’s cardiac arrest was CPVT.

This condition is estimated to have a prevalence of one per 10,000 persons [10]. CPVT is characterized by ventricular arrhythmias at elevated heart rates which present as syncope, palpitations, SCD, or SCA following an emotionally or psychologically stressful event [11-13]. CPVT has been connected with various mutations in the cardiac ryanodine receptor gene (RyR2) and calsequestrin 2 gene (CASQ2), which are inherited in an autosomal dominant or recessive pattern, respectively. Both of these genes' code for proteins involved in the regulation of calcium-induced release from the sarcoplasmic reticulum in cardiac myocytes [11-17]. This malfunction in regulation leads to increased diastolic calcium concentrations and therefore significant arrhythmias [10,17].

The clinical picture surrounding this patient's diagnosis of CPVT, including an emotional situation, past history of palpitations and chest pain, and family history of sudden cardiac death was classic and provided a basis for this patient to be diagnosed with CPVT and treated promptly. In up to 79% of patients eventually diagnosed with CPVT, the first presentation of symptoms is syncope, which as discussed above has a very broad differential diagnosis. In this population, diagnosis of CPVT was delayed by 2±0.8 years due to the assumption of vasovagal or neurological etiology [11-14].

CPVT is defined by studies such as Leenhardt et al. and Priori et al., which have established a characteristic presentation for this diagnosis. This includes the age of first symptoms of 3-17 years old, lack of EKG changes, normal cardiac imaging, family history of SCD, and cardinal symptom of syncope. This patient falls into the majority for all of these categories. In our extensive review, other published case reports of CPVT demonstrate patients who fall outside of the classic presentation. These features include increased or decreased age, EKG changes with long QT or T wave abnormalities, cardiac structural abnormalities including patent ductus arteriosus or atrial septal defects, symptoms present without sympathetic stimulation or lack of family history. Our patient exhibits all of the classic features of CPVT with syncope of cardiac etiology that resulted in cardiac arrest as his first presentation. This combination of classic characteristics and undeniable severity provided an ideal situation for his prompt diagnosis and treatment within one admission. This combination of all classic features and severe presentation has not been published to our knowledge.

We present this case to highlight the classic and most obvious presentation of CPVT, but also bring to light the rarity of a condition with a high mortality rate that is likely underdiagnosed. 92% of patients with CPVT were found to have a near-fatal or fatal event from ages 13-26 [18]. First symptoms of CPVT have been noted to present anywhere from age 2 to age 36, and therefore this condition should be considered for patients presenting beyond childhood and adolescence [11]. Symptoms can include SCA, SCD, palpitations, or syncope. CPVT does not necessarily have an abnormal baseline EKG or structural cardiac abnormalities and therefore can be differentiated from other etiologies causing similar symptoms including Brugada syndrome, long QT syndrome, valvular disease, or cardiomyopathies [6,11,19]. 33% of probands had a family history of sudden cardiac death or sudden cardiac arrest below the age of 40. The most characteristic feature of CPVT is that symptoms are induced by emotional or physiological stress [11-14].

Given significant mortality without treatment, CPVT should be considered and ruled out in patients presenting with any of the aforementioned symptoms or features. This evaluation should include referral to cardiology to confirm the clinical diagnosis with exercise and/or genetic testing. Exercise testing has been shown to have up to a 100% success rate at inducing ventricular arrhythmias in patients with CPVT. Interventions such as beta-blockers (e.g., nadolol and carvedilol) demonstrated a reduction in cardiac events in 30%-60% of patients, but the majority of patients do require ICD placement for complete control of arrhythmias [11,12,20]. In the ED, the most beneficial action for suspected CPVT is the termination of the arrhythmia if present, and prompt referral to cardiology for Holter monitoring, exercise testing, and genetic counseling as indicated.

## Conclusions

This case report demonstrates a classic presentation of CPVT. The symptoms, personal history, and family history provided a basis for prompt diagnosis and treatment of a rare condition with a high mortality rate. The majority of cases do not present in this outright manner but will frequently present with syncope. The most common cause of pediatric syncope is vasovagal and therefore misdiagnosis is common. In patients who present to the ED with a syncopal episode and fit any of the characteristics of CPVT (including syncope or cardiac arrest precipitated by stress, normal cardiac structure, normal QTc, and family history of sudden cardiac death) there should be a thorough evaluation and referral to cardiology to rule out a primary cardiac cause of the event.
